# Pathophysiology of cerebral small vessel disease: a journey through recent discoveries

**DOI:** 10.1172/JCI172841

**Published:** 2024-05-15

**Authors:** Nicolas Dupré, Antoine Drieu, Anne Joutel

**Affiliations:** 1 Université Paris Cité, Institute of Psychiatry and Neuroscience of Paris (IPNP), INSERM U1266, Paris, France.; 2 GHU-Paris Psychiatrie et Neurosciences, Hôpital Sainte Anne, Paris, France.

## Abstract

Cerebral small vessel disease (cSVD) encompasses a heterogeneous group of age-related small vessel pathologies that affect multiple regions. Disease manifestations range from lesions incidentally detected on neuroimaging (white matter hyperintensities, small deep infarcts, microbleeds, or enlarged perivascular spaces) to severe disability and cognitive impairment. cSVD accounts for approximately 25% of ischemic strokes and the vast majority of spontaneous intracerebral hemorrhage and is also the most important vascular contributor to dementia. Despite its high prevalence and potentially long therapeutic window, there are still no mechanism-based treatments. Here, we provide an overview of the recent advances in this field. We summarize recent data highlighting the remarkable continuum between monogenic and multifactorial cSVDs involving *NOTCH3*, *HTRA1*, and *COL4A1/A2* genes. Taking a vessel-centric view, we discuss possible cause-and-effect relationships between risk factors, structural and functional vessel changes, and disease manifestations, underscoring some major knowledge gaps. Although endothelial dysfunction is rightly considered a central feature of cSVD, the contributions of smooth muscle cells, pericytes, and other perivascular cells warrant continued investigation.

## Introduction

Cerebral small vessel disease (cSVD) is an umbrella term for a collection of distinct diseases with overlapping phenotypes caused by intrinsic lesions of intracranial vessels. cSVDs are commonly classified into sporadic and hereditary cerebral amyloid angiopathy (CAA) and cSVD distinct from CAA (1). The latter classification — a larger group of pathologies commonly related to aging, hypertension, or genetic factors — is the focus of this Review.

**Figure 1:**
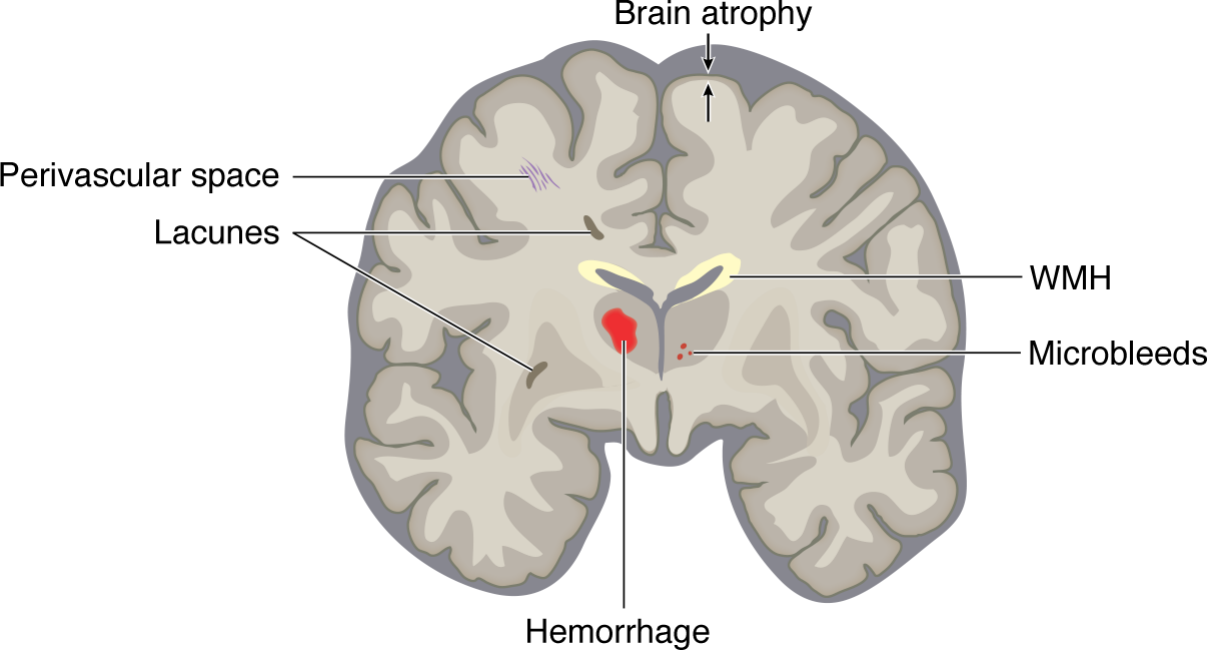
Neuroimaging features of cSVDs. In 2013, a group of experts published Standards for Reporting Vascular Changes on Neuroimaging (STRIVE-1) (2) — an attempt to harmonize terminology and definitions of key MRI features associated with cSVDs. These features include the following: white matter hyperintensities (WMHs) on T2-weighted MRI sequences (yellow); recent, small subcortical infarcts; subcortical lacunes of presumed vascular origin (3–15 mm fluid-filled cavities) (dark tan), likely the end result of a small subcortical infarct or microhemorrhage; perivascular (fluid-filled) spaces that follow the course of small perforating vessels (purple); microbleeds (2–5 mm diameter), detected as hypointense lesions on T2* images or susceptibility-weighted sequences (red); intracerebral hemorrhage (ICH) (red); and brain atrophy.

cSVDs are primarily defined by their hallmark features on brain MRI, including white matter (WM) hyperintensities (WMHs), small subcortical infarcts or lacunes, visible perivascular spaces (PVSs), microbleeds, intracerebral hemorrhage (ICH), and brain atrophy (Figure 1) (2). However, cSVD lesions detected by conventional MRI likely represent only the tip of the iceberg. Indeed, more sensitive imaging techniques such as diffusion tensor imaging (DTI) can detect altered diffusion properties in areas that appear normal on conventional MRI (3). The most common clinical manifestations associated with cSVDs include stroke, related to the occurrence of a small subcortical infarct; motor impairment; imbalance; and cognitive impairment. Cognitive deficits are predominantly characterized by altered executive functions and reduced processing speed (4). Other neuropsychiatric symptoms, such as apathy (a syndrome of reduced motivation), fatigue, depression, and delirium, are increasingly recognized as important features (5). The full spectrum of cSVD manifestations ranges from covert cSVD (brain lesions incidentally detected on brain MRI, especially in individuals 50–55 years or older with no overt clinical symptoms) to disability and dementia (6). Hence, cSVDs are thought to progress silently for many years before becoming clinically symptomatic, a conclusion supported by the natural history of monogenic forms that are largely indistinguishable from sporadic cSVDs (7).

cSVDs are among the most prevalent disorders that impact brain health at the population level, and their prevalence increases with age, affecting approximately 5% of those over 50 years old and almost everyone over 90 years old. Broadly speaking, cSVDs account for approximately 25% of ischemic strokes and the vast majority of spontaneous ICHs in aged individuals; they are also the second-most common cause of dementia after Alzheimer disease (4). Despite their high prevalence and potentially long therapeutic window, there are as yet no mechanism-based treatments for these devastating diseases. One major reason for this lack of treatment options is the complex multifactorial roots of cSVDs, which go well beyond blood clotting and vessel rupture.

Thanks to large-scale biomedical databases, international collaborative networks, and the affordability of high-throughput genotyping and sequencing, the past decade has witnessed major advances in our understanding of the genetic landscape of cSVDs. Technological developments in neuroimaging have enabled clinicians to probe functional abnormalities of small brain vessels in individual patients. Experimental studies, aided by newly developed models and cutting-edge imaging approaches, have identified novel mechanisms of vascular pathology in individual forms of cSVD as well as shared mechanisms among them. Taking a vessel-centric view, we summarize these advances and discuss the mechanisms linking structural and functional changes in brain vessels to disease manifestations. We conclude by highlighting some knowledge gaps and future perspectives.

## A continuum between monogenic and multifactorial cSVDs

In recent years, increasing numbers of genes have been associated with cSVDs (8–18). Notably, mutations in four genes — *NOTCH3*, *HTRA1* (high-temperature requirement A serine peptidase 1), *COL4A1* (collagen type IV α1), and *COL4A2* — account for the vast majority of monogenic adult-onset cSVDs (Table 1) (19–22). Among these monogenic forms, cerebral autosomal dominant arteriopathy with subcortical infarcts and leukoencephalopathy (CADASIL), caused by dominant missense mutations that alter the number of cysteines in one of the 34 EGF repeats in the extracellular domain of the NOTCH3 protein (NOTCH3^ECD^), is the most frequent (23). NOTCH3 is a transmembrane receptor predominantly expressed in mural cells — smooth muscle cells (SMCs) and pericytes — of small vessels. CADASIL mutations stereotypically lead to abnormal aggregation and accumulation of NOTCH3 and other extracellular matrix (ECM) proteins around mural cells, and cause pathology likely through a gain-of-function mechanism (24–26). Interestingly, recessive loss-of-function mutations in *NOTCH3* are associated with a rare and severe form of cSVD with a childhood onset (27–29). Pathogenic mutations in *HTRA1* can manifest as the rare recessive disease, cerebral autosomal recessive arteriopathy with subcortical infarcts and leukoencephalopathy (CARASIL), or a more frequent autosomal dominant cSVD through a loss-of-function or haploinsufficiency mechanism, respectively (10, 11). Notably, two different classes of pathogenic mutations have been identified in collagen type IV, producing radically different effects on collagen type IV expression and very different clinical presentations. Glycine-altering COL4A1/A2 variants produced by glycine substitutions within the triple-helical collagenous domain of COL4A1 or COL4A2 impair collagen IV folding and secretion into the basement membrane and manifest predominantly as spontaneous ICH in deep brain regions (30). In contrast, mutations within the 3′-untranslated region of *COL4A1* disrupt the binding site of the microRNA miR-29, resulting in increased COL4A1 expression. This in turn causes pontine autosomal dominant microangiopathy with leukoencephalopathy (PADMAL), an cSVD characterized by ischemic lesions (14).

Although monogenic cSVDs are thought to account for a small proportion (~5%) of cSVDs, variants in *NOTCH3*, *COL4A1/A2*, and *HTRA1* genes identical to those that cause monogenic cSVDs were recently found to be present at an unexpectedly high frequency in the general population and shown to increase the risk of stroke or dementia, with an additive interaction between cardiovascular risk factor burden and carrier status (Table 2) (31–39). The reason variants in these genes are associated with so broad a phenotypic spectrum is not yet fully understood. Nevertheless, for *NOTCH3* and *COL4A1/A2*, there is emerging evidence that the position of variants affects the penetrance and expressivity of disease manifestations (40–42). Furthermore, common variants near or in *NOTCH3*, *HTRA1*, or *COL4A1*/*A2* loci have been shown to be associated with cSVD features (Table 3) (8, 39, 43–51). In summary, these studies highlight a striking continuum between monogenic and multifactorial cSVDs. From an experimental perspective, these findings support the validity of genetically engineered animals carrying *Notch3*, *Col4a1*, *Col4a2*, or *Htra1* pathogenic variants as clinically relevant cSVD models.

## Structural and functional changes in cSVD brain vessels

Establishing the nature of structural and functional changes in brain vessels in cSVDs and the sequence and timeline linking these changes to brain lesions and clinical symptoms is fundamental to understanding the pathobiology of these complex diseases. Combining the complementary information gained from studies in patients and clinically relevant mouse models of cSVD is a powerful approach for illuminating these mechanisms.

## Vascular pathology

Brain arteriolosclerosis, a hallmark of cSVDs, affects small parenchymal arteries and arterioles and is defined by the degeneration and loss of SMCs, the concentric fibrohyalinotic (glassy-looking acellular) thickening of the arterial wall, the accumulation of ECM components, and subsequent narrowing of the lumen (52, 53). Arteriolosclerosis is highly prevalent in autopsy specimens from individuals over 70 years old, and its severity is significantly associated with the odds of lacunes, subcortical microinfarcts, and WM degeneration (54–56). Arteriole thrombosis and obliteration are only occasionally detected, although they are likely the cause of lacunar stroke (57). Arterial pathology is qualitatively similar between sporadic and hereditary cSVDs, but is quantitatively more aggressive in hereditary forms. In particular, degeneration and loss of arteriolar SMCs is especially severe in CADASIL and CARASIL patients (53). Although pericyte coverage was not specifically examined, a recent study reported a significant reduction in the number of capillary pericytes in the frontal deep WM, a region most frequently afflicted by cSVD, in postmortem tissues from patients with vascular dementia (58). Cerebral venules, which are often overlooked, can also display collagenosis (thickening of the walls with collagen), which has been associated with WM lesions (55, 56).

**Figure 2:**
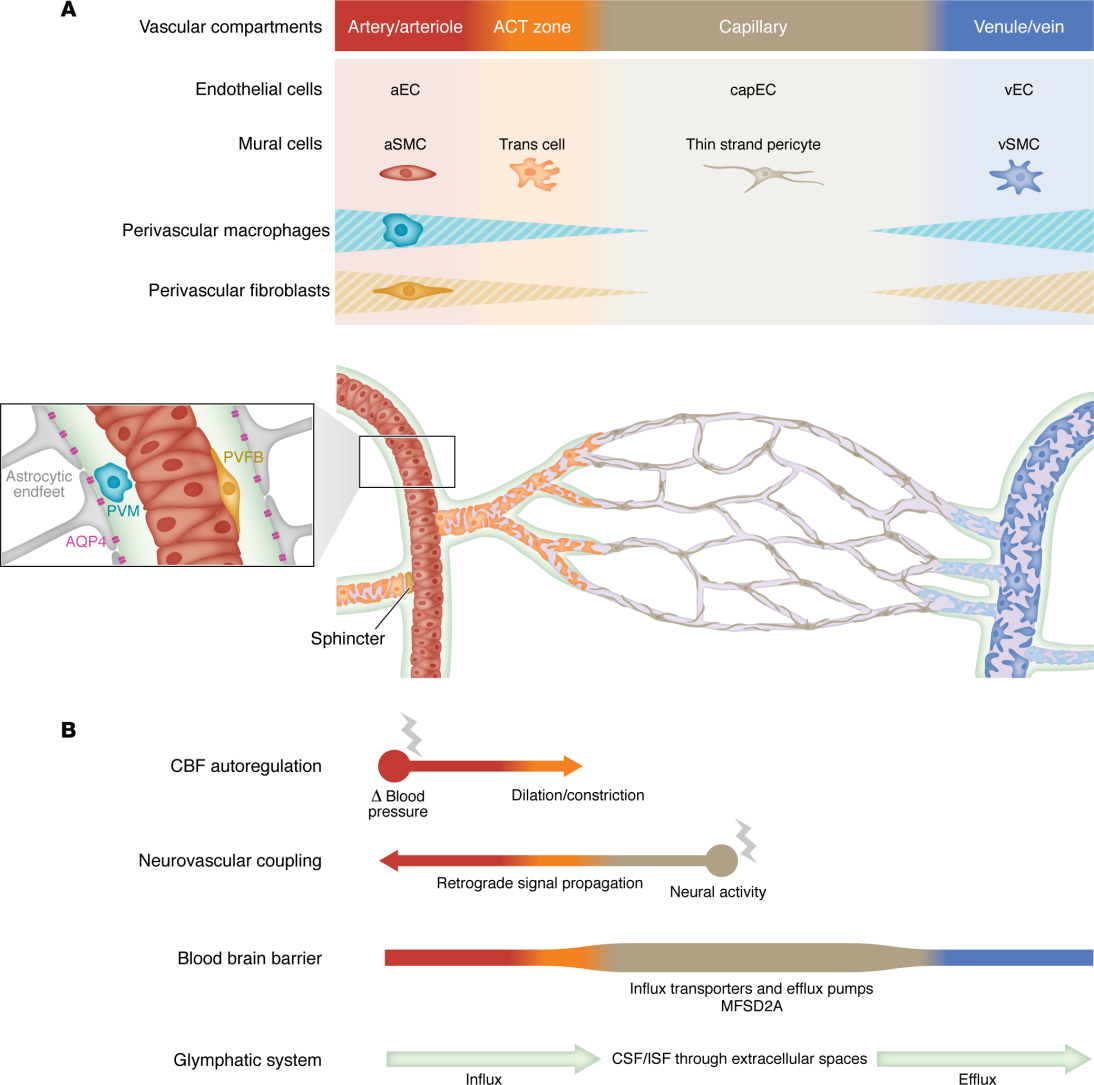
Integrated representation of the anatomy, cellular composition, and physiology of brain vessels. (**A**) Schematic of the arteriovenous axis with the four main vascular compartments, including the artery/arteriole, the arteriole-capillary transition (ACT) zone, the capillary bed and the venule/vein, and their associated cells: arterial endothelial cells (aECs), arterial SMCs (aSMCs), transitional cells (trans cells, orange), capillary endothelial cells (capECs), venous endothelial cells (vECs), and venous SMCs (vSMCs). Penetrating arteries and arterioles are separated from the brain parenchyma by a fluid-filled space (light green) that disappears as arterioles morph into capillaries and then reappears around veins. The perivascular space (inset) contains resident cells (PVMs and perivascular fibroblasts, PVFBs) and is delimited on the parenchymal side by the glia limitans formed by astrocytic endfeet. (**B**) Simplified depiction of the main brain vessel functions with respect to each vascular compartment. From top to bottom: (i) CBF autoregulation increases or decreases vessel diameter in response to BP decreases and increases, respectively. aSMCs are the primary sensors of BP changes and the primary effector cells driving changes in vessel diameter. (ii) Neurovascular coupling starts with the increase in local neural activity that leads to capEC hyperpolarization. Hyperpolarizing signal is propagated to upstream arterioles/arteries and transmitted to aSMCs, resulting in retrograde vasodilation. (iii) The BBB is formed by ECs, mural cells with their basement membrane, and astrocytic endfeet. Tight junctions between ECs prevent free paracellular transport of molecules; ECs express specific influx transporters and efflux pumps, which drive the active transport of specific solutes and metabolites into or out of the brain, respectively, and are enriched for the lipid transporter MFSD2A, which inhibits the rate of transcytosis (113, 166). (iv) The glymphatic system involves (a) CSF influx along the periarterial spaces, driven mainly by arterial pulsatility; (b) CSF entry into the brain supported by aquaporin 4 (AQP4) channel expression on the astrocytic endfeet, subsequent mix with the ISF, and flow through the extracellular spaces; and (c) the efflux of extracellular fluid and wastes along perivenous spaces.

**Figure 3:**
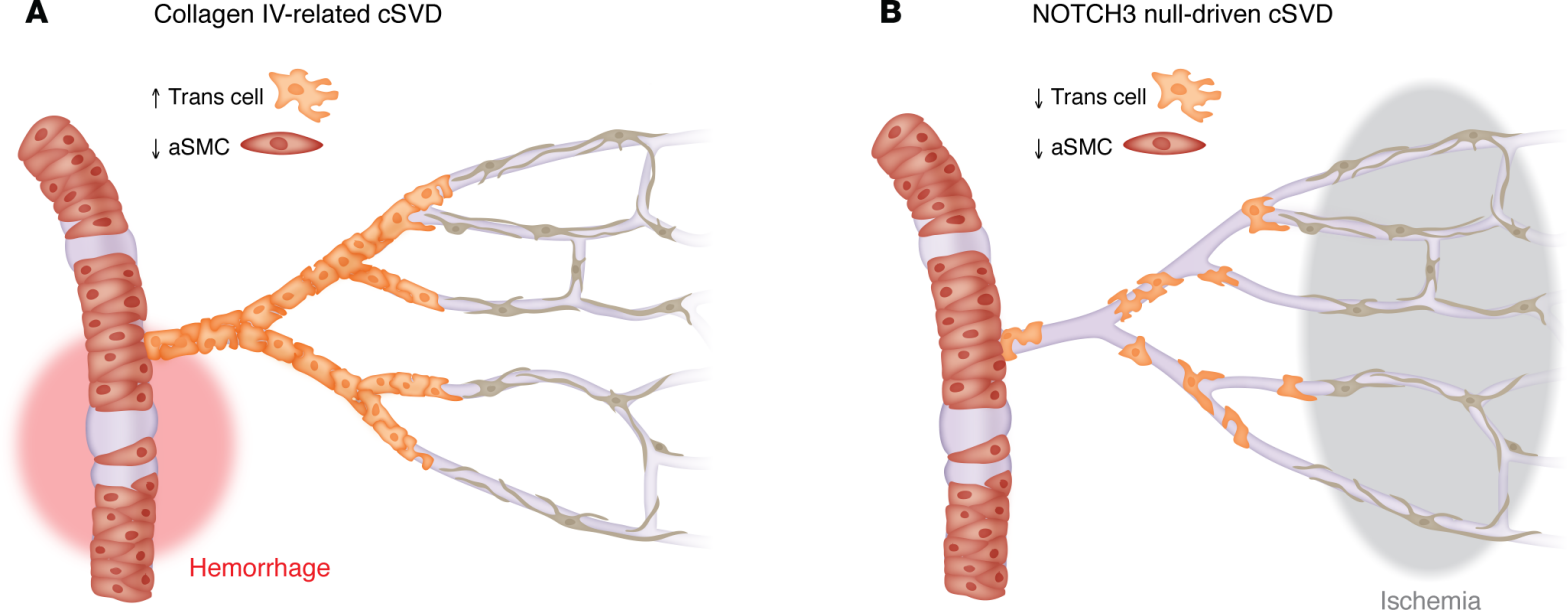
Opposite changes in mural cells in the arteriole-capillary transition zone are associated with distinct cSVD features. Schematic representation of brain vessels in (**A**) the collagen IV–related cSVD, which manifests as recurrent spontaneous ICHs, and in (**B**) the NOTCH3 null–driven cSVD, which is characterized by recurrent deep infarcts. (**A**) In the collagen IV disease, the ACT zone shows an increased number of mural cells with higher contractile protein content, raising intravascular pressure in the upstream feeding arteriole, which exhibits loss of SMCs, and promoting arteriolar rupture at the site of SMC loss and hemorrhage (red). (**B**) In the NOTCH3 null–driven cSVD, the combination of loss of arterial SMCs and loss of mural cells in the ACT zone is predicted to decrease perfusion pressure and promote ischemic lesions in the deep brain regions (gray).

Brain arteries of aged rodents exhibit increased tortuosity (59–61). Age-related focal loss and degeneration of arterial SMCs can be detected in the superficial vascular network of the retina, a developmental extension of the brain that enables robust quantification at cellular resolution thanks to its stereotypical and planar angioarchitecture (62). Extensive, early loss of arterial SMCs is a feature of brains and retinas of mice completely lacking *Notch3*, a model of a very severe form of human cSVD (63–65). Interestingly, a recent study suggested that age-related arterial SMC loss might be attributable to a decline in NOTCH3 signaling (66). The cerebroretinal vasculature of *Col4a1*-mutant mice expressing heterozygous missense mutations that substitute critical glycine residues (G498V, G1064D, or G1344D) within the triple helical domain of COL4A1 also exhibits arterial SMC loss (65, 67, 68). Patients and mice with *COL4A1* glycine-altering variants develop spontaneous ICHs in deep brain regions; importantly, pathological analyses of mutant mice indicate that ICHs originate from arteries with reduced SMC coverage, and demonstrate a strong correlation between ICH burden and the severity of arterial SMC loss (67). In contrast, spontaneous ICH is not observed in patients and mice completely lacking NOTCH3 protein, suggesting that loss of arterial SMCs is necessary but not sufficient to cause ICH (65). A major difference in vascular structural integrity between *Notch3*-KO and *Col4a1*-mutant mice resides at the level of the arteriole-capillary transition (ACT) zone that lacks an elastic lamina and is surrounded by contractile mural cells that possess more irregular ensheathing processes and a more rounded nucleus than SMCs (Figure 2) (65, 69–72). Here, *Notch3*-KO mice exhibit a loss of mural cells, whereas *Col4a1*-mutant mice show an increased number of mural cells in this zone with higher contractile protein content, a defect called “hypermuscularization.” Further genetic, functional, and computational modeling studies in *Col4a1*-mutant mice provided evidence that arteriole SMC loss and hypermuscularization of the ACT zone act as mutually reinforcing vascular defects to cause ICH, with the excessive ACT zone muscularization raising intravascular pressure in the upstream feeding arteriole and promoting arteriolar rupture at the site of SMC loss (Figure 3) (65). Molecular studies in *Col4a1*-mutant mice suggest that arterial SMC loss is driven by increased TGF-β activity, whereas the hypermuscularization of the ACT zone arises from increased NOTCH3 activity (65, 68). Regarding the capillary bed, 2D and 3D imaging in rodents revealed a small reduction in vascular length, branching density, and pericyte number, particularly in deep cortical layers and WM, in aged brains (59, 60). Pericyte coverage is reduced in *Htra1*-KO mice but, in striking contrast, pericyte density and/or coverage are preserved in *Notch3*-KO mice, *Col4a1*-mutant mice, and mice carrying an Arg169Cys mutation in NOTCH3 (hereafter referred to as CADASIL mice) (64, 67, 73, 74).

In summary, pathological changes can affect all microvascular compartments. Remarkably, however, structural defects can differ from one microvascular segment to another for a given cSVD and can differ between cSVDs for a given microvascular compartment. Loss and degeneration of arterial SMCs, which is often overlooked, is a key feature of cSVDs and not just an end-stage lesion. Loss of SMCs is especially prominent in severe cSVDs, suggesting that it is likely an important contributing mechanism.

## Vascular mechanics

Arteriolosclerosis is hypothesized to stiffen the arterial wall and reduce the ability of arteries to dilate. Using ultra-high-field (7T) quantitative flow MRI, two small case-control studies showed an increased pulsatility index in perforating arteries of the basal ganglia and the WM beneath the cortex in patients with sporadic cSVD or CADASIL compared with controls, suggestive of increased arterial stiffness (75, 76). Hypercapnia, likely through effects on both endothelial cells and SMCs, is a potent vasodilatory stimulus that is commonly used in clinics to assess the capacity of cerebral vessels to dilate (77). CO_2_-induced vasodilation is assessed by measuring cerebral blood flow (CBF) increases in response to breathing CO_2_ with functional MRI using blood O_2_ level–dependent (BOLD) response or arterial spin labeling, which can directly measure CBF. Two recent, large cross-sectional cohort studies showed that a reduced CO_2_ response in the WM or subcortical gray matter is associated with more severe cSVD burden (WMHs, lacunes, microbleeds, enlarged PVSs, and brain atrophy) and impaired cognition (78, 79). In a 1-year longitudinal study performed in patients with age-related WMHs, regions of normal-appearing WM that progress to WMHs over time had a lower baseline response to hypercapnia compared with normal-appearing WM (80).

In pharmacological and genetic models of hypertension, cross-sectional area and wall thickness are generally increased, which is considered an adaptive response to increased intravascular pressure that serves to reduce wall stress (81). These changes are associated with stiffening of large pial arteries, but increased distensibility of small pial arterioles. They also occur rapidly (within ~1 week) after the development of hypertension and can recover with blood pressure (BP) normalization (82). In angiotensin II–induced (AngII-induced) hypertensive models, a reduction in lumen diameter (inward remodeling) also develops slowly (over weeks) and does not recover with BP normalization (82). Inward remodeling is considered a maladaptive response to hypertension that is predicted to profoundly reduce CBF. Strikingly, pial arteries of CADASIL mice also exhibit inward remodeling, despite the fact that these mice are normotensive. This defect occurs very early, prior to any other functional changes (83, 84). Furthermore, *Htra1-*KO mice develop age-dependent accumulation of matrisome proteins, abnormal internal elastica lamina, and decreased distensibility at the level of pial arteries, again in the context of normal BP (74).

In summary, vessel wall remodeling and stiffening of large brain arteries appears to be a consistent feature across cSVDs, and these defects can occur very early in the disease process, even in a context of normal BP. Studies in patients suggest that the reduced capacity of brain vessels to dilate precedes the appearance of WMHs, one of the earliest types of damage in the brain parenchyma of cSVD patients. Moreover, this reduced dilatory capacity is correlated with the severity of cSVD brain lesions and thus may functionally contribute to them.

## CBF autoregulation

Autoregulation maintains relatively stable CBF in the face of moment-to-moment fluctuations in arterial BP. SMCs of arteries and arterioles are the primary sensors of changes in BP and the primary effector cells that drive intravascular pressure–dependent changes in diameter, increasing or decreasing vessel diameter in response to BP decreases and increases, respectively (Figure 2B) (85).

Dynamic CBF autoregulation (dCA) can be assessed in humans by quantifying how spontaneous fluctuations in BP are transferred to CBF from simultaneous recording of BP and CBF velocity using transcranial Doppler (85). A recent case-control study (*n =* 113 cSVD patients and 83 controls) showed that dCA was altered bilaterally in patients and that the degree of impairment was positively associated with the burden of cSVD MRI markers (86).

Experimental studies have shown that pressure-induced constriction (myogenic tone) and CBF autoregulation are impaired in hypertensive mice and in several models of monogenic cSVD. In young hypertensive mice, pial arteries exhibit increased myogenic tone and the autoregulation curve is right-shifted toward higher BP. However, this increase in myogenic constriction, which is thought to protect the distal portion of the vascular network from pressure overload, is lost in aged hypertensive mice (87). In mice carrying a G1344D or G394V glycine mutation in *Col4a1*, pial arteries exhibit an age-dependent reduction in myogenic tone caused by decreased activity of transient receptor potential melastatin 4 (TRPM4) channels, which are positive regulators of arterial tone (88, 89). Myogenic tone in *Col4a1*-mutant mice can be restored by improving COL4A1-COL4A2 trafficking using the chemical chaperone 4-phenylbutyrate (88). Interestingly, restoration of myogenic tone is associated with a reduction in the occurrence of ICH, an observation concordant with the current view that myogenic tone protects the vascular bed from pressure overload and ICH (88). In pial arteries of *Notch3-*KO mice, which exhibit arterial SMC loss, myogenic tone is strongly reduced and CBF autoregulation is severely compromised, with extreme narrowing of the autoregulated range (63, 90). In CADASIL mice, myogenic tone is reduced in pial arteries and penetrating arteries in the absence of overt SMC loss, and the lower limit of CBF autoregulation is right-shifted toward higher BP (83, 91). Decreased myogenic tone and impaired CBF autoregulation in CADASIL mice are attributable to the pathological accumulation of tissue inhibitor of metalloproteinase 3 (TIMP3) protein in NOTCH3^ECD^ deposits that results in increased density of voltage-gated potassium (K^+^) (K_V_) channels, which are powerful negative regulators of arterial tone, in arterial SMCs (92, 93).

In summary, there is some evidence that CBF autoregulation is compromised in patients and experimental models with cSVD, although the intrinsic mechanism — increased or decreased myogenic tone, loss or dysfunction of arterial SMCs — appears to differ among diseases. A key unanswered question is whether a rightward shift in the lower limit of CBF autoregulation to higher BP actually renders the brain, particularly its deep regions, more sensitive to low BP and its potential ischemic consequences.

## Resting CBF

Considering that WM receives its blood supply from the distal end of long medullary arteries (94), that WM lesions first start in brain areas that are furthest from the origin of the perforating arteries, and that brain arteriolosclerosis is characterized by stenosis of these arteries, it has long been thought that cSVD-related WM lesions are caused by chronic hypoperfusion. Consistent with this idea is the seminal observation of WM rarefaction in a mouse model of chronic hypoperfusion induced by bilateral common carotid artery stenosis (95). Numerous studies have explored the relationship between resting CBF and WMHs at the cross-sectional level. A recent meta-analysis including 2,180 participants from 34 studies showed that WMH burden in patients was worse and CBF was lower in regions with WMHs than in regions with normal-appearing WM. However, the few available longitudinal studies have yielded contradictory results (96); thus, whether reduction in resting CBF precedes or follows WMH progression remains in dispute. Concurrent measurement of the O_2_ extraction fraction (OEF), which can now be quantified using noninvasive MRI-based techniques, may be a promising approach for disentangling whether hypoperfusion is a cause or consequence of WMHs, since low CBF and elevated OEF are signatures of hypoxia/ischemia, whereas low CBF and low OEF indicate matched low O_2_ supply and demand (97).

Widespread reduction in resting CBF has been reported in *Notch3-*KO, CADASIL, and *Htra1*-KO mice, although the mechanism(s) are not fully understood (66, 74, 83). Interestingly, treatment with an AngII receptor type 1 (AT1) blocker improved CBF in *Htra1*-KO mice in association with reduced accumulation of matrisome proteins and amelioration of pial artery distensibility defects.

In summary, whereas chronic hypoperfusion is an indisputable feature in both cSVD mouse models and patients, whether it is a cause or consequence of brain damage and whether brain hypoperfusion contributes to disease manifestations remain unclear. In humans, recently developed approaches can disentangle this “chicken and egg” question. In mouse models, an in-depth characterization of brain lesions combined with a detailed analysis of the time course of CBF changes might be informative.

## Neurovascular coupling

Neurovascular coupling (NVC) — the ensemble of mechanisms that mediate activity-dependent increases in blood perfusion (functional hyperemia) — ensures appropriate delivery of nutrients and O_2_ in response to changes in local neural activity. Multiple redundant pathways and molecules are involved in linking neural activity to vessel dilation. A recent new paradigm envisions the vast capillary network within the brain acting as a sensory web capable of detecting increases in neuronal activity and sending rapid signals that dilate upstream arteries. In this conceptualization, extracellular K^+^ and nitric oxide (NO) are viewed as the most important neurovascular coupling mediators (98). Endothelial cells sense neural activity–derived K^+^ through the inward-rectifying K^+^ channel, Kir2.1, which is activated by modest elevations in extracellular K^+^ (produced during each action potential), resulting in endothelial cell hyperpolarization. This hyperpolarizing signal rapidly propagates retrogradely from cell to cell through the capillary network via gap junctions, ultimately reaching upstream arterioles and pial arteries. There, the signal passes to SMCs through myoendothelial projections, dilating arteries/arterioles and increasing blood flow to the site of signal initiation (Figure 2B) (99). More distally located thin-strand pericytes have also been reported to regulate capillary blood flow, but with slower kinetics than arteriolar SMCs and mural cells of the ACT zone (100). Besides supplying the metabolic needs of active neurons, NVC may serve additional purposes, such as removing metabolic waste through a vascular route, homogenizing flow in the capillary network, preventing capillary stalls by leucocytes, regulating brain temperature, facilitating cerebrospinal fluid (CSF) movement, and stabilizing the vascular network (101).

NVC can be assessed in humans by monitoring responses to a motor or visual stimulus using functional MRI to measure BOLD responses or through application of arterial spin labeling techniques. But despite the availability of such approaches, there are few studies on cSVD patients. Two independent case-control studies reported significant changes in NVC in CADASIL patients, demonstrating reduced amplitude or a time-shifted decrease in the hemodynamic response (75, 102). However, one caveat with human studies is that the altered blood flow response might be related to a reduction in the neural response due to brain lesions rather than a decrease in NVC efficiency originating in the vascular bed.

Mouse studies have pointed to vascular rather than neural causes of NVC deficiencies. Among these, one recent report provided convincing evidence that aging-related deterioration is caused by an age-dependent decrease in vasoresponsivity that is most pronounced at precapillary sphincters (a novel structure identified in the cortex at the transition between some arterioles and the ACT zone; ref. 103), rather than caused by reduced neuronal activity (104). Hypertension also impairs NVC (105). In AngII-induced chronic hypertension, activation of AT1 receptors in perivascular macrophages (PVMs, a population of resident macrophages in the PVS) is involved in NVC deficits and leads to the production of reactive oxygen species, which impair endothelium-dependent responses (106). NVC is also disrupted in the BPH/2 mouse model of neurogenic hypertension (107). The underlying mechanisms involve PVMs, as described above, as well as defective capillary-to-arteriole signaling caused by a diminished activity of the capillary endothelial cell Kir2.1 channel (108). Strikingly, *Col4a1*-mutant and CADASIL mice, like hypertensive mice, exhibit an age-dependent reduction in functional hyperemia that also results from defective capillary-to-arteriole signaling as a consequence of diminished capillary endothelial cell Kir2.1 channel activity. Remarkably, the fundamental defect underlying this channelopathy (depletion of the minor membrane phospholipid phosphatidylinositol 4,5-bisphosphate (PIP_2_), a key activator of the Kir2.1 channel) is similar in *Col4a1*-mutant and CADASIL mice, although the intrinsic mechanisms differ (25, 88, 92, 93, 109–111). Interestingly, restoring functional hyperemia by depleting PVMs in hypertensive mice and by chronic inhibition of phosphoinositide-3-kinase (PI3K) in *Col4a1*-mutant mice improved memory deficits (106, 111). Although these findings support the hypothesis that a chronic reduction in NVC could account for cognitive deficits, further studies are needed to substantiate this relationship and rule out possible confounding effects of specific experimental maneuvers, which may have additional effects on the brain or brain vessels.

In summary, deterioration of NVC is a recurrent theme in mouse models of sporadic and genetic cSVDs. Remarkably, experimental studies have identified shared mechanisms between sporadic and genetic cSVDs, pointing to dysfunction of a single endothelial cell ion channel (Kir2.1) in both cases. Studies in CADASIL patients and mouse models of CADASIL suggest that NVC is impaired prior to the development of subcortical infarcts (93, 102). Additional human studies are needed to assess the reproducibility of BOLD responses in cSVD patients (112) and study the timeline of NVC dysfunction with respect to the appearance of brain lesions and clinical manifestations. Further experimental studies are warranted to better understand whether and how chronic dysfunction of such an important mechanism impairs brain functioning.

## Regulation of fluids in the brain

The blood-brain barrier (BBB) limits the movement of ions, amino acids, molecules, and cells into and out of the brain (Figure 2B) (113). The glymphatic system is a fluid-clearance pathway thought to primarily serve the function of nonselectively clearing metabolic waste from the brain interstitial space (114). This process, which is primarily active during sleep, is initiated by the flow of CSF along the PVS-surrounding arteries and its entry into the brain. CSF mixes with the interstitial fluid (ISF) in the parenchyma, leaves the brain along perivenular spaces, and is ultimately exported by meningeal lymphatic vessels and along cranial and spinal nerve sheaths toward the cervical lymph nodes (Figure 2B) (114, 115). The flow of CSF in the spaces around pial arteries is pulsatile, reflecting dynamic changes in arterial diameter caused by cardiac impulse waves (arterial pulsatility), which are important physiological drivers that pump CSF inward along these spaces (116). Recent work has further implicated PVMs as important regulators of CSF flow dynamics through their involvement in arterial motion and remodeling of the vascular ECM (117).

Leakage of fibrinogen and other plasma proteins into the brain parenchyma has harmful effects that affect microglial activation, cause neuronal and axonal loss, and promote demyelination and inhibition of remyelination (118, 119). Moreover, this leakage can increase interstitial fluid and cause WM edema. Two studies, using diffusion MRI and a 2-compartment model, support the possibility of increased extracellular free water in WM in patients with sporadic cSVD or CADASIL (120, 121). On the basis of these observations, it has been proposed that cSVD-related brain lesions (WMHs or infarcts) could arise from a leaky BBB. Alternatively, Benveniste and Nedergaard recently crafted the novel hypothesis that failure of fluid transport via the glymphatic system could cause enlargement of PVSs, accumulation of interstitial fluid in WM, and ultimately, demyelination (122).

BBB integrity in humans can be assessed by quantifying the dynamic extravasation (paracellular leakage) of small contrast agent molecules (~550 Da for gadolinium) into the brain parenchyma using dynamic contrast-enhanced MRI. Cross-sectional case-control studies have generally shown changes consistent with widespread, but subtle (i.e., detectable after noise filtering) BBB leakage as well as hotspots of increased BBB permeability in patients with sporadic cSVD (123–125). Cohort studies have shown an association between WMH volume and increased BBB permeability (126, 127). Moreover, longitudinal studies have identified a link between BBB leakage at baseline and the loss of microstructural integrity over time in the perilesional zones around WMHs and further showed that greater BBB leakage at baseline was associated with more severe decline in cognitive functions, especially executive function. Taken together, these data suggest that BBB impairment might play an early role in subsequent WM lesions (128, 129). Nonetheless, studies in CADASIL patients have produced contradictory results (125, 130). Analyzing water exchange across the BBB using arterial spin labeling MRI is another promising approach for assessing subtle BBB dysfunction since water’s molecular weight (~18 Da) is much smaller than that of gadolinium-based contrast agents (131, 132). Using arterial spin labeling MRI, Yang and colleagues recently reported a diffuse alteration (in the whole brain) in the water exchange rate across the BBB in patients with CADASIL or HTRA1-related cSVD, suggestive of an increase in the BBB’s permeability to water (133). BBB integrity in the mouse is compromised upon aging (134). It has also been reported that AngII-induced hypertension enhances BBB permeability by reducing endothelial tight junctions and increasing transcytosis, mainly in arterioles and venules. The mechanism underlying this enhanced BBB permeability primarily involves cooperative interactions of AT1-expressing endothelial cells with PVMs (135). In contrast, the BBB was reported to be preserved in the deoxycorticosterone acetate salt hypertensive model, as well as in adult *Col4a1*-mutant, CADASIL, or CARASIL mice (67, 73, 74, 136).

How might these human and mouse BBB studies be reconciled? One possibility is the presence of widespread, but subtle, BBB dysfunction in cSVDs, mainly manifesting as increased permeability to water and ions; permeability to water has not yet been assessed in mouse models. On the other hand, a number of confounding factors that could mimic or mask BBB leakage can affect dynamic contrast-enhanced MRI measurements (137). Moreover, hotspot sites of BBB leakage may reflect the presence of recent microinfarcts (73).

DTI along the PVS (DTI-ALPS) around deep medullary veins is an emerging noninvasive technique for evaluating the glymphatic system in humans. The ALPS index represents the CSF efflux function along the perivenous spaces, although additional validation studies are needed (138). Three recent studies performed in population-based cohorts or in patients with sporadic cSVD showed that the DTI-ALPS index was negatively related to the presence and severity of cSVD MRI markers (WMH, lacunes, microbleeds, visible PVS in the basal ganglia, and brain atrophy), suggestive of a declined glymphatic function (139–141). Another small case-control study reported a lower DTI-ALPS index in CADASIL patients compared with controls and an association between the DTI-ALPS index and disease (neuroimaging and clinical) severity (142). However, one potential limitation of these studies is that the DTI-ALPS index is derived from the DTI signal, which is particularly sensitive to WM damages in cSVD and that none of these studies controlled for conventional DTI measures. Studies in mice have shown that aging is associated with progressive glymphatic system dysfunction, manifesting as reductions in both glymphatic influx and efflux. Two factors are primarily responsible for reduced glymphatic influx: decreased arterial wall compliance, which reduces the perivascular pumping of CSF, and depolarization of aquaporin 4, which decreases the transport of fluid across astrocytic endfeet into the brain (143). Glymphatic efflux is likely reduced because of a decrease in the number and diameter of meningeal lymphatics (144). Acute hypertension strongly slows CSF influx in PVSs by increasing backflow (116) and glymphatic transport, both influx and efflux, is altered in spontaneously hypertensive rats (145). A recent study suggested a reduction in glymphatic influx in *Notch3-*KO mice, possibly because of decreased contractility of cerebral arteries or a reduced number of PVMs (66, 146).

In summary, emerging evidence suggests that the glymphatic system is compromised in cSVD and that impaired CSF/ISF dynamics may participate in the pathogenesis, but this warrants further studies.

## Concluding remarks and future directions

Studies in patients and experimental models suggest that functional vascular changes appear years (in humans) or weeks/months (in mice) after exposure to risk factors, but before the appearance of brain lesions. These changes may include stiffening of large arteries, a reduction in dilation capacity and blood flow, attenuation of NVC, subtle BBB leakage, and glymphatic system dysfunction. Nevertheless, much work is still needed to finely map the time course of these changes and assess the causal relationships between these changes and disease manifestations. Going forward, studies in patients with monogenic cSVDs may overcome the confounding issue of heterogeneity of patients with sporadic cSVDs, who may simultaneously display comorbidities and other neurodegenerative processes. It is also worth noting that research to date has tended to focus on WM lesions, with small subcortical infarcts and lacunes receiving less notice, despite the fact that lacune count is a strong predictor of disability and cognitive impairment (147, 148).

Although experimental studies have highlighted candidate mechanisms, elucidating the mechanistic chains linking risk factors, vascular changes, and brain lesions remains a daunting challenge. A first issue to clarify is which cell type(s) are involved and at what steps of the pathogenic process. The endothelial cell is the cornerstone in the regulation of CBF and BBB, and endothelium-dependent functions, such as NVC, are affected early during aging and hypertension or in the setting of pathogenic variants. On the basis of these observations, it has been proposed that cSVDs are initiated in the endothelium and that endothelial dysfunction is a key driver of vascular pathology (149). However, mural cells are also critical effector cells of major brain vessel functions and are certainly key contributors that must not be overlooked. In particular, human and rodent studies indicate that loss of arterial SMCs is a common denominator in many cSVDs, a linkage underscored by the observation that the most aggressive cSVD forms are associated with more severe loss of arterial SMCs. Several recent studies have also highlighted PVMs and perivascular fibroblasts, another major resident cell of PVS, as additional important candidate cellular mediators. A second question pertains to the molecular pathways that lead to vascular cell loss or dysfunction. Genetic, molecular, proteomic, and functional studies point to an important role for proteins of the microvascular ECM as a convergent mechanism in cSVDs (8, 150). Identifying shared molecular pathways among cSVDs would trigger a quantum leap in the development of therapeutic strategies. Another issue relates to the respective contributions of different microvascular compartments. The observation that the ACT zone is hypermuscularized in ICH-related cSVDs and less muscularized in cSVDs with a mostly ischemic presentation suggests that changes in the properties or density of mural cells in this segment could influence the hemorrhagic versus ischemic presentation of cSVDs (Figure 3). Therefore, in-depth molecular, structural, and functional characterizations of each microvascular compartment in distinct cSVD models could potentially provide insight into the relationship between vessel changes and brain damage.

As noted above, cSVDs are especially prevalent with aging. cSVDs and neurodegenerative diseases share similar risk factors and often co-occur. Consequently, other neurodegenerative pathologies can contribute to the clinical presentation. Another major challenge will thus be to clarify whether these two pathologies progress independently of each other or exhibit synergistic interactions.

In conclusion, cSVDs have an enormous impact on human health. Fortunately, the field is now advancing rapidly. Large collaborative research networks that bring together complementary expertise from basic science laboratories to clinics dedicated to cSVDs should enable further breakthroughs in the near future, bringing us closer to the development of therapeutics that can slow the progression of these devastating diseases.

## Figures and Tables

**Table 3:**
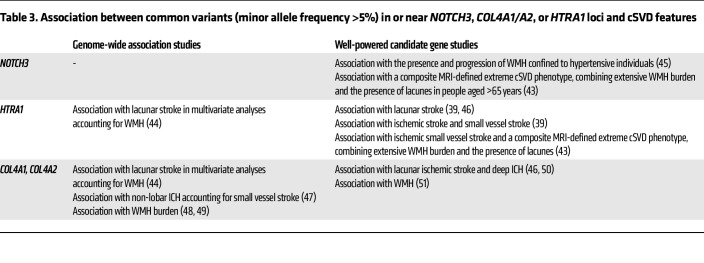
Association between common variants (minor allele frequency >5%) in or near *NOTCH3*, *COL4A1/A2*, or *HTRA1* loci and cSVD features

**Table 2:**
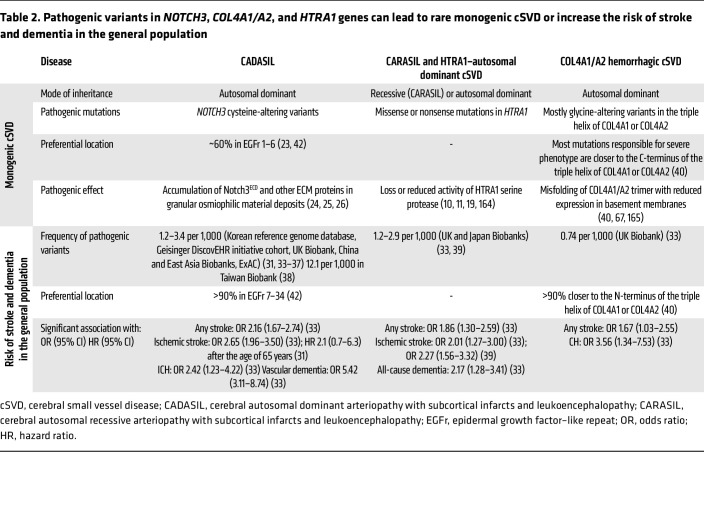
Pathogenic variants in *NOTCH3*, *COL4A1/A2*, and *HTRA1* genes can lead to rare monogenic cSVD or increase the risk of stroke and dementia in the general population

**Table 1:**
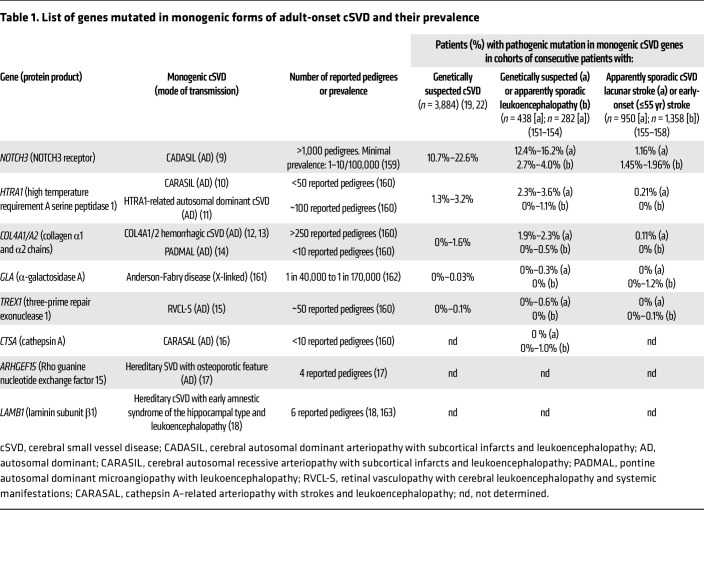
List of genes mutated in monogenic forms of adult-onset cSVD and their prevalence

## Version 1 05/15/2024

Electronic publication
